# Supplementary material for: Seizure likelihood varies with day-to-day variations in sleep duration in patients with refractory focal epilepsy: A longitudinal electroencephalography investigation
Source: eClinicalMedicine. 2021 Jun 5;37:100934. doi: 10.1016/j.eclinm.2021.100934 (PMC8343264; doi:10.1016/j.eclinm.2021.100934)
Supplement: Supplementary file 1 [file mmc1.docx]

# Supplementary Material

**Supplementary Table 1.** **Results of repeated measures one-way ANOVAs looking at the effects of current sleep-wake state on seizure rate**

| Patient # | df | F | p-value | Tukey’s multiple comparisons test | Significant differences in seizure rate |
| --- | --- | --- | --- | --- | --- |
| 1 | 5 | 0∙66 | p=0∙50 | -- | -- |
| 2 | 5 | 0∙82 | p=0∙38 | -- | -- |
| 6 | 5 | 0.92 | p=0∙34 | -- | -- |
| 8 | 5 | 12∙38 | p<0∙0001 | p<0.0001 | ↑Awake vs NREM2 |
|  |  |  |  | p<0∙0001 | ↑Awake vs NREM3 |
|  |  |  |  | p<0∙0001 | ↑Awake vs REM |
|  |  |  |  | p=0∙029 | ↓NREM3 vs NREM2 |
|  |  |  |  | p=0∙037 | ↓NREM3 vs NREM1 |
|  |  |  |  | p=0∙001 | ↑WASO vs NREM2 |
|  |  |  |  | p<0∙0001 | ↑WASO vs NREM3 |
|  |  |  |  | p=0∙0032 | ↑WASO vs REM |
|  |  |  |  |  |  |
| 9 | 5 | 6∙35 | p=0∙011 | p<0∙0001 | ↑Awake vs NREM3 |
|  |  |  |  | p<0∙0001 | ↑Awake vs REM |
|  |  |  |  | p=0∙029 | ↑Awake vs WASO |
| 10 | 5 | 2∙59 | p=0∙054 | -- | -- |
| 11 | 5 | 36∙60 | p<0∙0001 | p<0∙0001 | ↑NREM1 vs Awake |
|  |  |  |  | p<0∙0001 | ↑NREM1 vs NREM2 |
|  |  |  |  | p<0∙0001 | ↑NREM1 vs NREM3 |
|  |  |  |  | p<0∙0001 | ↑NREM1 vs REM |
|  |  |  |  | p<0∙0001 | ↑NREM1 vs WASO |
|  |  |  |  | p=0.013 | ↑NREM2 vs NREM3 |
| 12 | 5 | 0∙88 | p=0∙35 | -- | -- |
| 13 | 5 | 1∙08 | p=0∙30 | p<0∙0001 | ↑REM vs Awake |
|  |  |  |  | p=0∙0007 | ↑REM vs NREM3 |
|  |  |  |  | p=0.0075 | ↑NREM2 vs NREM3 |
|  |  |  |  | p=0.0017 | ↑NREM2 vs Awake |
| 15 | 5 | 1∙33 | p=0∙25 | -- | -- |

**Supplementary Table 2. Results of one-way ANOVAs looking at the effects of current sleep-wake on seizure duration.**

| Patient # | df | F | p-value |
| --- | --- | --- | --- |
| 1 | 5 | 1∙70 | p=0∙15 |
| 2 | 5 | 0∙47 | p=0∙80 |
| 6 | 5 | 0.81 | p=0∙55 |
| 8 | 5 | 0∙55 | p=0∙74 |
| 9 | 5 | 0∙76 | p=0∙58 |
| 10 | 5 | 2∙19 | p=0∙054 |
| 11 | 4 | 1∙56 | p=0∙19 |
| 12 | 3 | 1∙59 | p=0∙29 |
| 13 | 5 | 1∙90 | p=0∙092 |
| 15 | 5 | 0∙29 | p=0∙91 |

**Supplementary Table 3. Results of mixed effects Logistic Regression models**

| Logistic Model | Risk Factor | Risk of seizure  Odds ratio relative to baseline (95% Confidence interval) | *p*-value  (Bonferroni corrected α=0.25) | Change in the risk of seizure relative to baseline |
| --- | --- | --- | --- | --- |
| Total Sleep Duration | ↓ sleep | 0∙92 (0∙74, 1∙15) | 0**∙**48 | -- |
|  | ↑ sleep | 0∙73 (0∙58, 0∙93) | **0∙01** | ↓ |
|  | Age | 1∙01 (0∙90, 1∙15) | 0∙83 | -- |
|  | Gender | 2∙81 (0∙28, 27∙93) | 0∙38 | -- |
|  | EZ PT vs OP | 0∙84 (0∙017, 40∙98) | 0∙93 | -- |
|  | EZ PT vs T | 0∙50 (0∙012, 21∙57) | 0∙72 | -- |
|  | EZ PT vs FT | 1∙53(0∙026, 87∙34) | 0∙84 | -- |
|  | EZ OP vs T | 1∙68 (0∙16, 17∙84) | 0∙67 | -- |
|  | EZ OP vs FT | 0∙55 (0∙059, 5∙07) | 0∙60 | -- |
|  | EZ T vs FT | 0∙33 (0∙031, 3∙41) | 0∙35 | -- |
| Proportion WASO | ↓ WASO | 1∙02 (0∙81, 1∙27) | 0∙89 | -- |
|  | ↑ WASO | 0∙87 (0∙69, 1∙09) | 0∙22 | -- |
|  | Age | 1∙01 (0∙90, 1∙15) | 0∙82 | -- |
|  | Gender | 2∙95 (0∙30, 28∙89) | 0∙35 | -- |
|  | EZ PT vs OP | 0∙81 (0∙17, 38∙84) | 0∙92 | -- |
|  | EZ PT vs T | 0∙49 (0∙11, 20∙85) | 0∙71 | -- |
|  | EZ PT vs FT | 1∙45 (0∙026, 80∙83) | 0∙85 | -- |
|  | EZ OP vs T | 1∙67 (0∙15, 17∙27) | 0∙68 | -- |
|  | EZ OP vs FT | 0∙56 (0∙061, 5∙13) | 0.61 | -- |
|  | EZ T vs FT | 0∙34 (0∙033, 3∙51) | 0.37 | -- |
| Proportion NREM1 | ↓ NREM1 | 0∙91 (0∙72, 1∙14) | 0∙43 | -- |
|  | ↑ NREM1 | 0∙97 (0∙78, 1∙22) | 0∙82 | -- |
|  | Age | 1∙01 (0∙89, 1∙15) | 0∙82 | -- |
|  | Gender | 2∙95 (0∙302, 28∙95) | 0∙35 | -- |
|  | EZ PT vs OP | 0∙82 (0∙017, 38∙99) | 0∙92 | -- |
|  | EZ PT vs T | 0∙50 (0∙012, 20∙94) | 0∙71 | -- |
|  | EZ PT vs FT | 1∙46 (0∙026, 81∙15) | 0∙86 | -- |
|  | EZ OP vs T | 1.65 (0∙16, 17∙28) | 0.68 | -- |
|  | EZ OP vs FT | 0∙56 (0∙061, 5∙13) | 0.61 | -- |
|  | EZ T vs FT | 0.34 (0∙033, 3∙51) | 0.37 | -- |
| Proportion NREM2 | ↓ NREM2 | 1∙04 (0∙84, 1∙31) | 0∙68 | -- |
|  | ↑ NREM2 | 1∙01 (0∙80, 1∙27) | 0∙93 | -- |
|  | Age | 1∙01 (0∙90, 1∙15) | 0∙82 | -- |
|  | Gender | 2∙97 (0∙30, 29∙01) | 0∙35 | -- |
|  | EZ PT vs OP | 0∙82 (0∙17, 38∙79) | 0∙92 | -- |
|  | EZ PT vs T | 0∙49 (0∙12, 20∙82) | 0∙71 | -- |
|  | EZ PT vs FT | 1∙45 (0∙026, 80∙60) | 0∙86 | -- |
|  | EZ OP vs T | 1∙65 (0∙16, 17∙28) | 0∙68 | -- |
|  | EZ OP vs FT | 0∙56 (0∙061, 5∙13) | 0∙61 | -- |
|  | EZ T vs FT | 0∙34 (0∙033, 3∙51) | 0∙37 | -- |
| Proportion NREM3 | ↓ NREM3 | 0∙79 (0∙63, 1∙00) | 0∙050 | -- |
|  | ↑ NREM3 | 0∙92 (0∙74, 1∙15) | 0∙45 | -- |
|  | Age | 1∙01 (0∙90, 1∙15) | 0∙82 | -- |
|  | Gender | 2∙95 (0∙30, 28∙94) | 0∙35 | -- |
|  | EZ PT vs OP | 0∙82 (0∙017, 39∙09) | 0∙92 | -- |
|  | EZ PT vs T | 0∙49 (0∙012, 20∙88) | 0∙71 | -- |
|  | EZ PT vs FT | 1∙46 (0∙026, 81∙56) | 0∙85 | -- |
|  | EZ OP vs T | 1.66 (0∙16, 17∙28) | 0∙67 | -- |
|  | EZ OP vs FT | 0.56 (0∙061, 5∙13) | 0∙61 | -- |
|  | EZ T vs FT | 0.34 (0∙033, 3∙51) | 0∙36 | -- |
| Proportion REM | ↓ REM | 1∙29 (1∙03, 1∙63) | 0∙025 | -- |
|  | ↑ REM | 1∙26 (1∙01, 1∙57) | 0∙035 | -- |
|  | Age | 1∙01 (0∙90, 1∙14) | 0∙82 | -- |
|  | Gender | 2∙98 (0∙30, 29∙35) | 0∙35 | -- |
|  | EZ PT vs OP | 0∙81 (0∙017, 38∙95) | 0∙92 | -- |
|  | EZ PT vs T | 0∙49 (0∙02, 20∙97) | 0∙71 | -- |
|  | EZ PT vs FT | 1∙44 (0∙026, 80∙41) | 0∙86 | -- |
|  | EZ OP vs T | 1∙64 (0∙16, 17∙29) | 0∙68 | -- |
|  | EZ OP vs FT | 0∙57 (0∙061, 5∙19) | 0∙62 | -- |
|  | EZ T vs FT | 0∙34 (0∙033, 3∙56) | 0∙37 | -- |


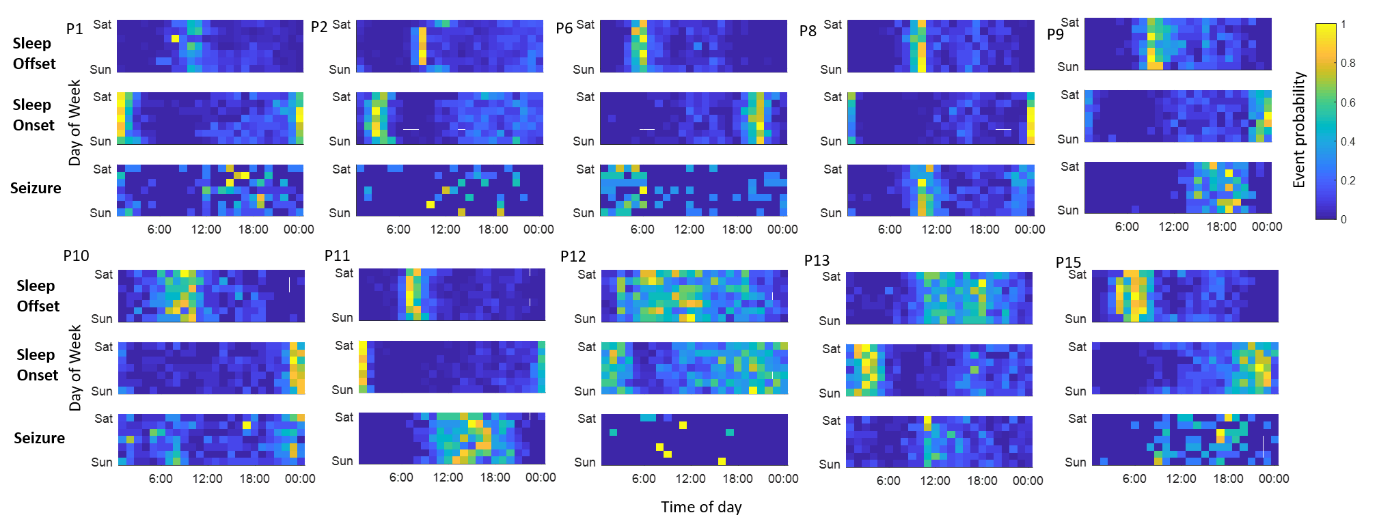


**Supplementary Figure 1.** **Circaseptan cycle of seizure, sleep onset and sleep offset.** The probability of sleep offset (top), sleep onset (middle) and seizure (bottom) are indicated for each patient. Yellow indicates a higher probability of the event and blue a low probability.


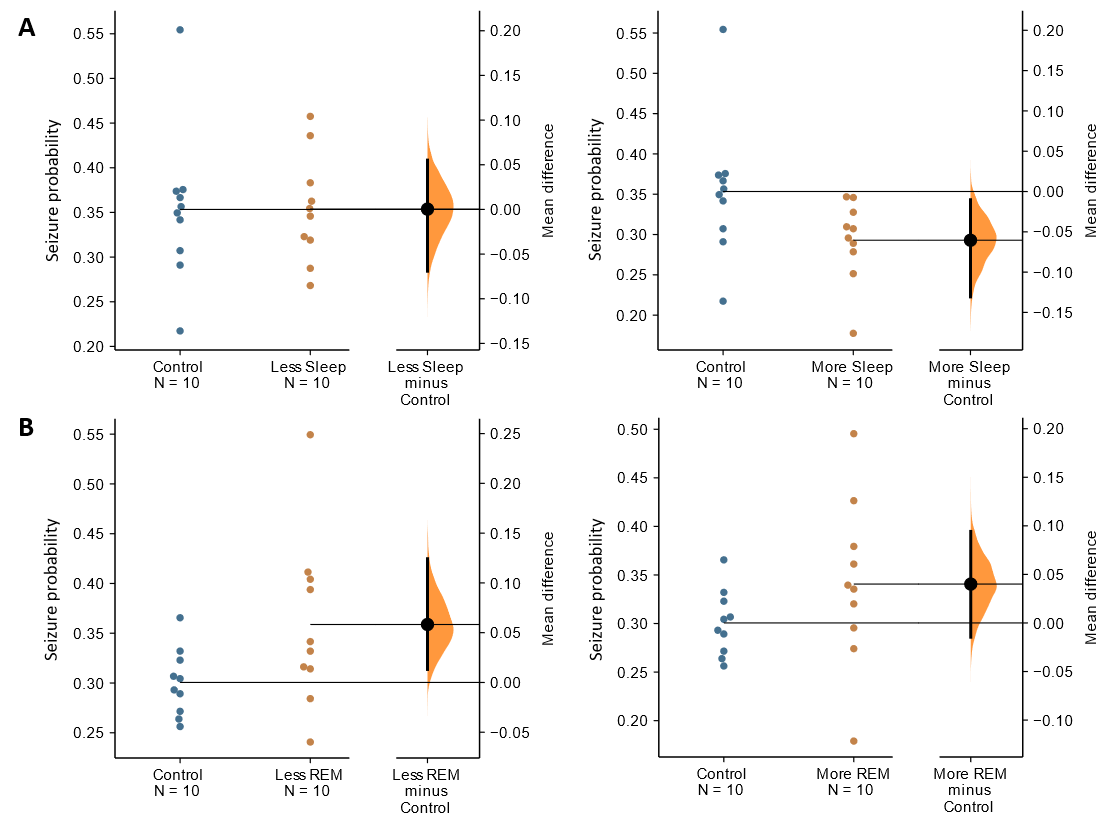


**Supplementary Figure 2.** **Seizure probability following changes in sleep duration and REM proportion.** The Gardner-Altman^1^ estimation plots demonstrate the difference between the means for A (left) control and less sleep, A (right) control and more sleep, B (left) control and less REM and B (right) control and more REM. For each figure the patient seizure probabilities are plotted on the left axes (coloured dots); the mean difference is plotted on a floating axis on the right as a bootstrap sampling distribution (5000 samples; bias corrected and accelerated). The mean difference is depicted as a dot; the 95% confidence interval is indicated by the ends of the vertical error bar.

# References

1. Ho J, [Tumkaya](javascript:;) T, [Aryal](javascript:;) S, et al. Moving beyond p-values: data analysis with estimation graphics. *Nature Methods* 2019; **16:** 565-566
